# Cough-induced rib fracture in a smoker: a case report

**DOI:** 10.1186/s13256-020-02497-4

**Published:** 2020-09-05

**Authors:** Aimee Daccache, Jad Haddad, Ahmad Ghanem, Elio Junior Feghali, Bassel El Osta

**Affiliations:** 1 Gilbert and Rose-Marie Chagoury School of Medicine, Blat, Lebanon; 2 Mazloum Hospital, Tripoli, Lebanon

**Keywords:** Cough, Rib fracture, Chest pain

## Abstract

**Background:**

Coughing is considered an important mechanism that helps the body get rid of foreign substances or prevent their entry into the tracheobronchial tree. Infrequently, after the onset of coughing, patients presenting with persistent chest pain are found to have rib fractures. Among the cases reported for cough-induced rib fractures, the maximum number of fractured ribs was found to be four.

**Case presentation:**

In this report, we present a case of a healthy 50-year-old Lebanese smoker who developed a total of six fractures in five ribs, asymmetrically, after coughing for 2.5 months.

**Conclusion:**

This case report is, to our knowledge, the first to describe six cough-induced rib fractures in a smoker without an underlying predisposition.

## Introduction

Coughing is considered an important mechanism that helps rid the body of foreign substances or prevent their entry into the tracheobronchial tree [1]. Coughing is often self-limited and uncomplicated. However, when severe, it can be associated with pneumothorax, pulmonary herniation, or rib fractures [2].

**Table 1:** Table of laboratory test values from Dr. Zahia Chahine (26 Jan 2018). Tripoli: Nini Hospital

Parameter	Value (normal range)
CRP	18.4 mg/L (0.0–5.0 mg/L)
ESR	30 mm (0–15 mm)
25-OH vitamin D (total)	6.7 ng/ml (30–100 ng/ml)
TSH	0.7372 IU/ml (0.47–4.64 IU/ml)
T3	0.81 ng/ml (0.79–1.49 ng/ml)
T4	7.13 g/dl (4.5–12.0 g/dl)
CEA	1.90 ng/ml (0–5.0 ng/ml)
PTH	50.51 pg/ml (15–68 pg/ml)
AST	20 U/L (5–35 U/L)
ALT	23 U/L (6–45 U/L)
Serum creatinine	0.85 mg/dl (0.5–1.4 mg/dl)
PSA	1.293 ng/ml (0–4 ng/ml)

*Abbreviations: ALT* alanine aminotransferase, *AST* aspartate aminotransferase, *CEA* carcinoembryonic antigen, *CRP* C-reactive protein, *ESR* erythrocyte sedimentation rate, *PSA* prostate-specific antigen, *PTH* parathyroid hormone, *TSH* thyroid-stimulating hormone

**Fig. 1:**
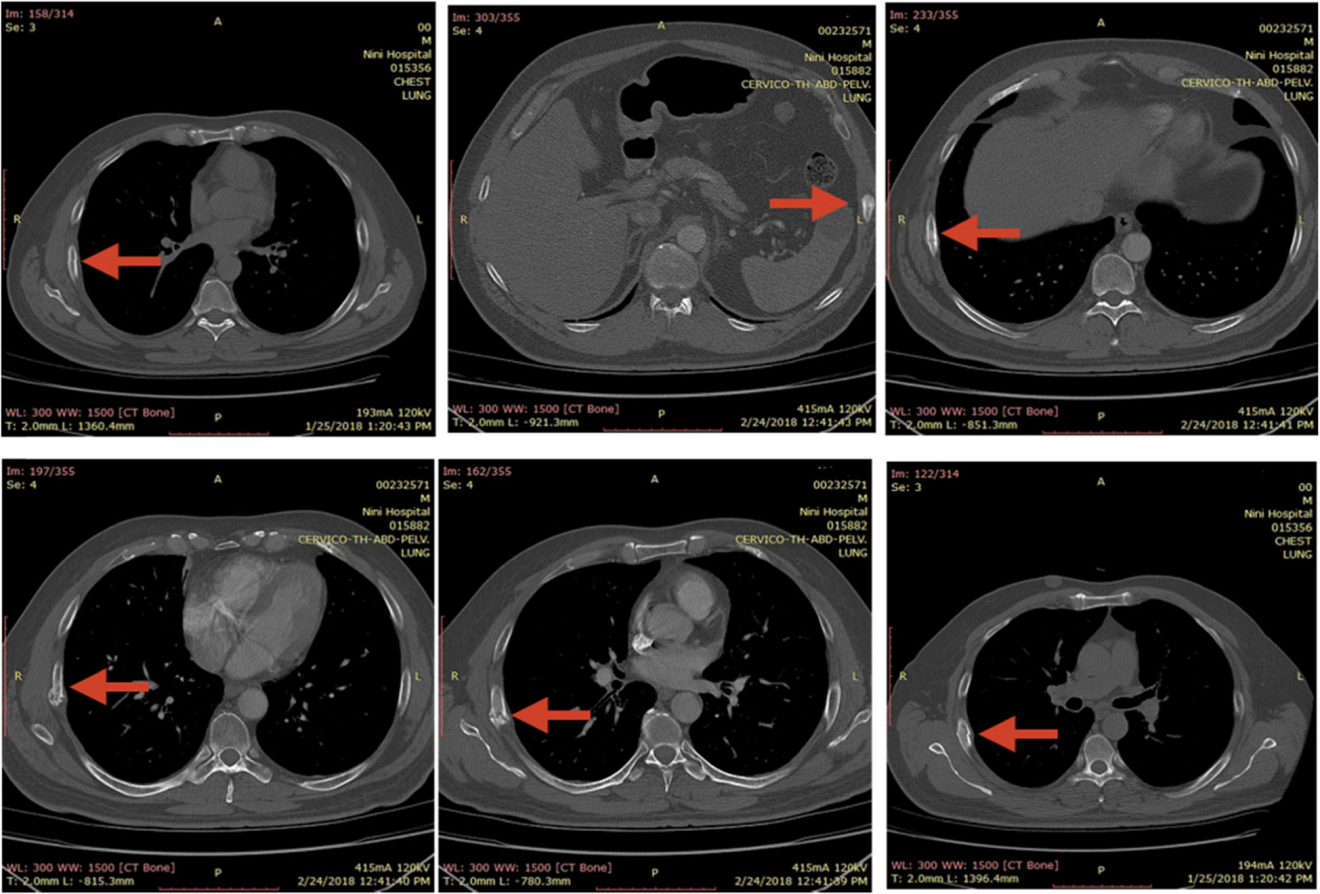
Chest computed tomographic scan shows fractures of fifth, sixth, seventh, and ninth left ribs and the anterior bow of the fifth right rib *(red arrow)*. Dr. Bassel El Osta (25 Jan 2018), Tripoli: Nini Hospital

**Fig. 2:**
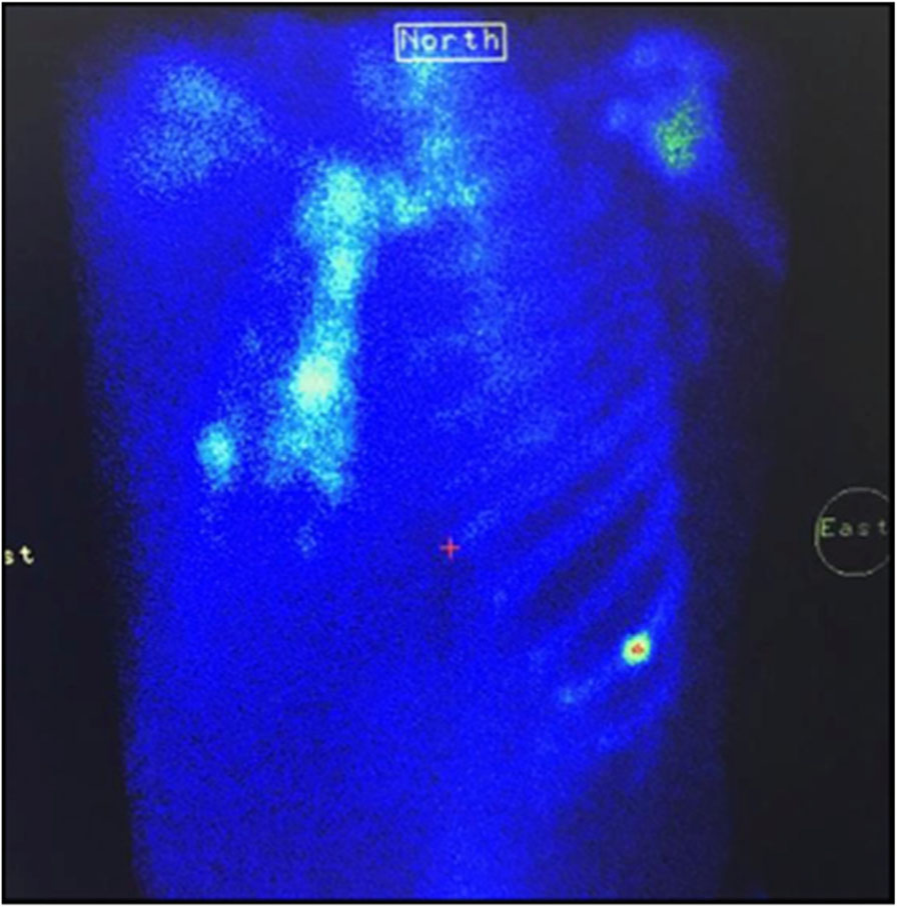
Bone scintigraphy showing multiple foci of bone hyperfixation at the anterior arch of the ninth left side; average arches of the fifth, sixth, and seventh straight ribs (aligned foci); and the anterior bow of the fifth right rib (double fracture). Dr. Bassel El Osta (25 Jan 2018), Tripoli: Nini Hospital

Most commonly, rib fractures are caused by a thoracic injury. Infrequently, after the onset of coughing, patients presenting with persistent chest pain are found to have rib fractures [3].

Previous studies showed that cough-induced rib fractures occurred most frequently on the lateral side of the fifth through ninth ribs [1], with the sixth rib being the most common site [4]. Among the cases reported for cough-induced rib fractures, the maximum number of fractured ribs was found to be four [3].

In this report, we present a case of an otherwise healthy 50-year-old patient who developed a total of six fractures in five ribs, asymmetrically, after coughing for 2.5 months. The uniqueness of this report is its description of the oddly large number of rib fractures involved in a healthy smoker with no relevant history of diseases. This case report may prompt attention to the importance of addressing the issue of cough-induced rib fracture and of dealing with chronic cough to avoid such complications.

## Case presentation

A 50-year-old Lebanese man presented to our hospital for ongoing chest pain of 2.5 months’ duration felt upon coughing, sneezing, or laughing. The patient had experienced a mild episode of self-limited bronchitis 3 months ago for 1 week, after which he developed constant, dry, nonproductive cough and chest pain. In the patient’s past medical history, chest trauma, chronic diseases, and allergies to any medication were absent. With regard to his social and professional history, the patient is a wealthy businessman who works in Saudi Arabia. He does not consume alcohol at all. He does not take any drug for any chronic or acute disease. He has no history of cancer or similar condition in his family. His physical examination revealed no remarkable findings except for tenderness upon palpation of the chest, mainly in the midaxillary line. No crackle, wheeze, or barrel chest was present. His pulse was slightly increased from baseline, reaching 86 beats/minute due to pain. His blood pressure was 130/85 mmHg. His body temperature was normal at 36.2 °C. A complete blood count and a metabolic panel were ordered to rule out any anemia or plasma cell dyscrasia, and the results of these were normal. Blood tests ordered and performed were the following: prostate-specific antigen, thyroid function test (thyroid-stimulating hormone, T3, and T4), carcinoembryonic antigen, parathyroid hormone, liver function tests, and serum creatinine. His blood tests showed no abnormal findings except for a low vitamin D level. In addition, his erythrocyte sedimentation rate and C-reactive protein were found to be elevated (Table 1). Additional workup was needed. Abdominopelvic, neck, and chest computed tomographic (CT) scans (Fig. 1) were ordered first to rule out any metastatic, prostate, or focal cancers. His lung parenchyma and bronchial wall thickness were normal. Following the CT scans, an x-ray showed fractures of the lateral right sides of the sixth and seventh ribs. Because the patient was a smoker, a bone scintigraphy scan (Fig. 2) was performed in order to rule out any metastatic disease. Several foci of bone hyperfixation were identified by scintigraphy. The findings were distributed as follows: (1) anterior arch of the ninth left rib; (2) anterior arches of the fifth, sixth, and seventh right ribs creating aligned foci; and (3) double fractures in the anterior bow of the fifth right rib. Other bone abnormalities were insignificant at this stage. No pulmonary function tests were done, because the patient did not show any signs of chronic obstructive pulmonary disease. Finally, his osteodensitometry results were normal.

The patient was managed with antitussives and nonsteroidal anti-inflammatory drugs, with which he slowly improved. For his chest pain, the patient was administered simple analgesia consisting of codeine phosphate and acetaminophen, with tramadol if needed, and the patient did not need it. Three months later, the patient completely recovered. At his 6-month follow-up, the patient was symptom-free with no chest pain and with a complete normal examination with no pain in his ribs. At his 9-month of follow-up, the patient was completely well, so no further investigations were done.

## Discussion

We report a case of a 50-year-old male smoker, a businessman who presented to our hospital for ongoing chest pain with no associated relevant medical history except a 1-week history of self-limited bronchitis approximately 3 months earlier followed by a persistent cough. Following examination and imaging, a total of six cough-induced rib fractures, distributed on both sides, were recorded, which makes it the highest number of cough-induced rib fractures in an otherwise healthy smoker reported to date, to our knowledge.

It has been proposed that cough-induced rib fractures happen due to strong bending forces leading to small cracks in the ribs. Another mechanism is the serratus anterior and external oblique muscles that generate shearing forces on the middle one-third of the rib [5]. In a study done by Hanak *et al.* [3], four patients were reported to have five or more fractured ribs without detailing the exact number, knowing that the remaining patients were specified to have one, two, three, or four fractures at most.

It was reported that cough-induced rib fractures appear more commonly in women than in men [4]. In addition, rib fractures occur normally with chronic cough following either respiratory system infections, chronic obstructive pulmonary disease, or bronchial asthma under steroid therapy. These were concluded to be risk factors for cough-induced rib fracture [6].

It was reported that cough-induced rib fractures occurred most frequently on the lateral side of the fifth through ninth ribs [1], with the sixth rib being the most commonly fractured site [3]. Fracture locations in our patient were the anterior arch of the ninth left rib and the anterior arches of the fifth, sixth, and seventh right ribs, creating aligned foci and double fractures in the anterior bow of the fifth right rib, thus in compliance with previously reported data.

## Conclusion

Our patient’s case is, to our knowledge, the first to involve six cough-induced rib fractures in a single healthy patient without an underlying predisposition. The only potential risk factor recorded in our patient was smoking one pack of cigarettes per day for the past 10 years. Rib fracture locations were in compliance with previously reported data. Physical examination and plain radiography frequently miss costal arch fractures if no bone pathology or history of trauma is present. Any delay in diagnosis increases the chances of further complications, including chronic pain and rupture of organs. New-onset chest pain with a background of chronic cough makes cough-induced rib fracture a probable differential diagnosis.

## Data Availability

All data generated or analyzed during this study are included in this published article.

## References

[1] Sünnetçioğlu A, Batur A. Cough-induced rib fracture: a case report. East J Med. 2015;20(4):254–5.

[2] Trovato DA, Sousa JE, Bruetman JE, Finn BC, Young P. Symmetrical rib fractures associated with chronic cough: report of one case [in Spanish]. Rev Med Chil. 2018;146(3):391–3. 10.4067/s0034-98872018000300391 29999111

[3] Hanak V, Hartman T, Ryu J. Cough-induced rib fractures: review of 54 cases [abstract]. Chest. 2005;128(4 Suppl):294S.

[4] Sano A, Tashiro K, Fukuda T. Cough-induced rib fractures. Asian Cardiovasc Thorac Ann. 2015;23(8):958–60. 10.1177/0218492315596893 26207003

[5] Yeh CF, Su SC. Cough-induced rib fracture in a young healthy man. J Formos Med Assoc. 2012;111(3):179–80. 10.1016/j.jfma.2011.07.020 22423673

[6] Sakellaridis T, Andrianopoulos E, Stamatelopoulos A, Laoutides G, Kormas P. Cough induced rib fractures in patients with respiratory infection. Chirurgia. 2008;21(2):73–6.

